# MicroRNAs in the Myelodysplastic Syndrome

**DOI:** 10.32607/actanaturae.11209

**Published:** 2021

**Authors:** Y. A. Veryaskina, S. E. Titov, I. B. Kovynev, S. S. Fedorova, T. I. Pospelova, I. F. Zhimulev

**Affiliations:** Institute of Cytology and Genetics, SB RAS, Novosibirsk, 630090 Russia; Institute of Molecular and Cellular Biology, SB RAS, Novosibirsk, 630090 Russia; Vector-Best, Novosibirsk, 630117 Russia; Novosibirsk State Medical University, Novosibirsk, 630091 Russia

**Keywords:** myelodysplastic syndrome, miRNA, acute myeloid leukemia

## Abstract

The myelodysplastic syndrome (MDS) holds a special place among blood cancers, as it represents a whole spectrum of hematological disorders with impaired differentiation of hematopoietic precursors, bone marrow dysplasia, genetic instability and is noted for an increased risk of acute myeloid leukemia. Both genetic and epigenetic factors, including microRNAs (miRNAs), are involved in MDS development. MicroRNAs are short non-coding RNAs that are important regulators of normal hematopoiesis, and abnormal changes in their expression levels can contribute to hematological tumor development. To assess the prognosis of the disease, an international assessment system taking into account a karyotype, the number of blast cells, and the degree of deficiency of different blood cell types is used. However, the overall survival and effectiveness of the therapy offered are not always consistent with predictions. The search for new biomarkers, followed by their integration into the existing prognostic system, will allow for personalized treatment to be performed with more precision. Additionally, this paper explains how miRNA expression levels correlate with the prognosis of overall survival and response to the therapy offered.

## INTRODUCTION

MicroRNAs (miRNAs) are short non-coding RNAs that regulate gene expression post-transcriptionally. To date, more than 2,600 human miRNAs have been identified, each with the potential to regulate hundreds of target genes [1]. MicroRNAs play key regulatory roles in all biological processes, including cell proliferation, cellular differentiation, cell cycle control, apoptosis, and angiogenesis [2, 3, 4, 5]. In addition, miRNAs can act as either oncogenes or as suppressors of tumors of various origins, including hematological malignancies [6, 7]. MicroRNAs are important regulators of the differentiation and maintenance of hematopoietic stem cells (HSCs), and changes in their expression levels obviously promote the development of myeloid and lymphoid neoplasms [8].

Myelodysplastic syndromes (MDSs) are a heterogeneous group of HSC disorders characterized by bone marrow cell dysplasia and a deficiency of one or more blood cell types that have to do with inefficient hematopoiesis [9]. Although no epidemiological data on MDS have been gathered yet in Russia, the Surveillance, Epidemiology, and End Results (SEER) states that the incidence of the disease in the U.S. was above 28,032 in 2012–2017, with the majority of patients being above 70 years of age [10]. In addition, MDS presents an increased risk of transformation into acute myeloid leukemia (AML) [11]. The mechanisms of MDS initiation and development are not yet fully understood, and the current methods of treatment and diagnosis are not sufficiently efficient, since the disease comes in many facets [12].

Since information about the role of miRNAs in MDS development and prognosis is fragmentary, this overview considers the role miRNAs play in normal hematopoiesis and provides a comprehensive analysis of the variations in their expression levels in MDS patients with normal and aberrant karyotypes. Special attention is given to the examples portraying miRNAs as promising markers for predicting the development of MDS and the effectiveness of the therapy offered.

## MicroRNA BIOGENESIS

The discovery of the small non-coding RNA *lin-4 *in* Caenorhabditis elegans *in 1993 laid the foundation for a new line of research. The main finding of that discovery was the fact that *lin-4 *downregulates the* lin-14 *gene post-transcriptionally by complementarily binding the unique short miRNA sequence of *lin-4 *(the seed region) to the 3’ untranslated region (3’-UTR) of the *lin-14 *gene [13, 14]. According to PubMed, about 100,000 articles reporting on the results of miRNA studies have been published to date. Some miRNA genes are located within other genes, mostly in the introns and occasionally in the exons of protein-coding genes, but many more are located in intergenic regions and are regulated by their own promoters, which are similar to those of protein-coding genes. However, transcript processing in the former is more complex [15].

**Fig. 1:**
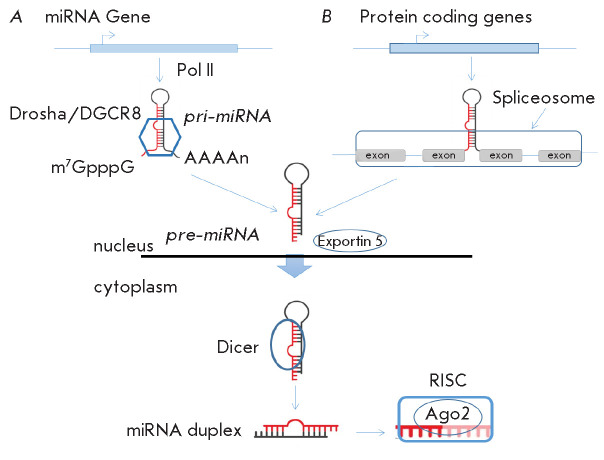
MicroRNA processing. (*A*) – Canonical pathway. MicroRNAs are transcribed by RNA polymerase II to produce primary transcripts (pri-miRNAs). The pri-miRNAs are cleaved by a microprocessor that includes Drosha and DGCR8 to form precursor miRNAs (pre-miRNAs). The pre-miRNAs are exported from the nucleus to the cytoplasm by the protein Exportin 5. Dicer cleaves the loop, and one strand of the miRNA duplex binds to the Ago2 protein to form the RNA-induced silencing complex (RISC). (*B*) – One of the non-canonical pathways for miRNA processing (Drosha-independent). Splicing results in the formation of short intron hairpins to become a substrate for further miRNA processing

**Fig. 2:**
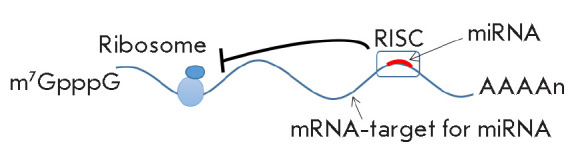
Mechanism of mRNA translational repression by the miRNAs incorporated into a RISC, which includes mature miRNAs. The RISC interferes with the ribosome advancing along the mRNA and, thus, stops the translation

miRNA is 18-24 nucleotides in length, and its formation is a multistep process involving a large number of enzymes: DNA → primary miRNA (pri-miRNA) → precursor miRNA (pre-miRNA) → mature miRNA. The miRNA biogenesis pathway can be either canonical or non-canonical (*Fig. 1*). The canonical pathway is more common, and if miRNA biosynthesis follows it, pri-miRNAs are transcribed by RNA polymerase II (Pol II) and converted into pre-miRNAs by a complex consisting of the RNA-binding protein DGCR8 and the enzyme Drosha. Next, a hairpin RNA ~70 nt in length (pre-miRNA) is exported to the cytoplasm by a complex consisting of the Exportin5/RanGTP proteins. Then, the endonuclease Dicer removes the terminal loop, resulting in a mature miRNA duplex. Because alternative strands can be differentially represented in different tissues, mature miRNAs are often suffixed “5p” or “3p” to denote the functional miRNA strand. Normally, the strand with the lower 5’ stability or with the 5’ uracil is called ‘the guide strand’ and eventually becomes incorporated into the RNA-induced silencing complex (RISC) directly involved in the regulation of target genes, while the other strand, called ‘the passenger strand’, is removed (*Fig. 2*). The target gene is silenced by mRNA cleavage at 10–1 nt upstream of the 5’-end of the guide strand. This cleavage is mediated by the activity of the Ago2 protein to be one of the main components of the RISC complex [16].

cases of the processing of certain miRNAs. In particular, the processing of miRNA-451, which holds an important place in hematopoiesis, follows this non-canonical pathway. Primary miRNA-451 is processed by enzymes in the Drosha/DGCR8 complex, and the resulting pre-miRNA-451 directly binds to the Ago2 protein, the main component of the RISC complex [18]. Relatively rare non-canonical pathways are not considered in detail in this overview.

In most cases, miRNAs interact with the 3’-UTR of target mRNAs; however, interactions of miRNAs with other regions, including the 5’-UTR, the coding sequence, and gene promoters, have also been reported [16]. Mature miRNAs largely interact with target mRNAs due to complete or partial complementary binding of the seed region of the miRNAs to the 3’-UTR of the target mRNAs. It should be noted that binding with imperfect complementarity is possible, because a single miRNA can target multiple genes [19]. The degree of complementarity determines what will take place: Ago2-dependent cleavage of the target mRNA or translational suppression [16]. Currently, there are information resources (miRTarBase, TargetScan, mirDB, miRWalk, miRanda) that allow one to predict the genes targeted by miRNAs and, thus, identify the most specific miRNAs for the disease under study. However, to understand the role of miRNA in the mechanisms of initiation and development of blood diseases, comprehensive knowledge of miRNA functions in maintaining normal hematopoiesis is required.

## MicroRNAs IN NORMAL HEMATOPOIESIS

Hematopoiesis is a process of blood cell formation that begins in early embryogenesis and appears as a cascade of divisions and differentiation of hematopoietic stem cells (HSCs) [20]. Stem cells undergo symmetric and asymmetric division, and, thus, their population is maintained and differentiated cells form. Symmetric division implies the formation of two identical cells, while asymmetric division results in the formation of one initial and one differentiated cell, the latter being capable of making it all the way from a multipotent precursor to a mature blood cell. Multipotent precursors (MPPs) produce a common lymphoid progenitor (CLP) and a common myeloid progenitor (CMP). MDSs are a group of diseases of hematopoietic stem cells and are characterized by multilineage dysplasia in immature myeloid cells and ineffective hematopoiesis. Impaired myeloid cell development is the primary cause of MDS [21].

**Fig. 3:**
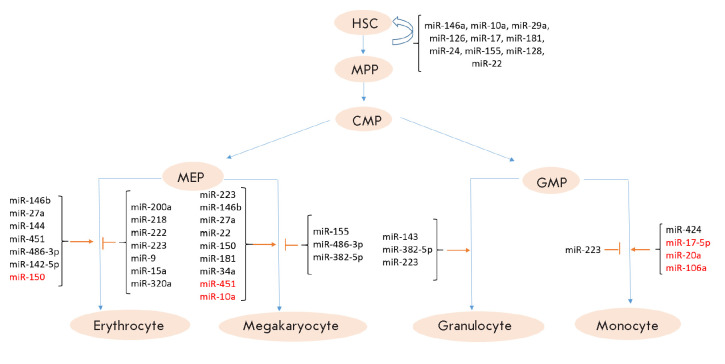
A Schematic of myelopoiesis with a list of the miRNAs involved in the regulation of various stages of normal hematopoiesis. The names of miRNAs with increased expression levels are typed in black, and k; the names of those with decreased expression levels during hematopoiesis regulation – in red

Both genetic and epigenetic regulatory mechanisms, including miRNAs, are involved in the maintenance of normal hematopoiesis (*Fig. 3*). Chen *et al. *published one of the first works describing the role of miRNAs in the differentiation control of hematopoietic lineages in mammals [22]. According to many studies, miRNAs are involved in the regulation of all branches of the hierarchical tree of blood cell development [23, 24]. Importantly, mutations of the *Dicer *gene, whose product is a key participant in miRNA processing, affect normal hematopoiesis, suggesting that miRNA regulation plays an important role in it [25, 26]. The balance between the self-renewal and differentiation of stem cells is also controlled by miRNAs. Georgantas *et al.* name 33 miRNAs specific to hematopoiesis; in particular, miRNA-17, -24, -146, -155, -128 and -181, which block HSC differentiation into more mature blood cells [27]. MicroRNA-22 is yet another player in the control of HSC self-renewal [28]. Gupta *et al*. emphasize the point that the expression levels of miRNA-146a, -10a, -29a, -126, -17, and miRNA-181 are increased in HSCs and that their function is to maintain the HSC phenotype and to regulate the transition of MPPs to CMPs or CLPs [8]. It was further established on mouse cells that miRNA-125a, -99, -130a, and miRNA-33 are involved in the control of HSC self-renewal [26, 29, 30, 31].

A MDS diagnosis is based on the identification of dysplastic changes in at least one hematopoietic lineage. Morphological changes in the cells involved in erythro-, granulocyto- and megakaryocytopoiesis in the bone marrow and blood are very diverse, and the ratio of normal to dysplastic elements varies significantly from one patient to another. A cell line is considered to be modified if its dysplastic elements amount to more than 10%. Now, let us consider the role of miRNAs in the development of each hematopoietic lineage in more detail.

**Fig. 4:**
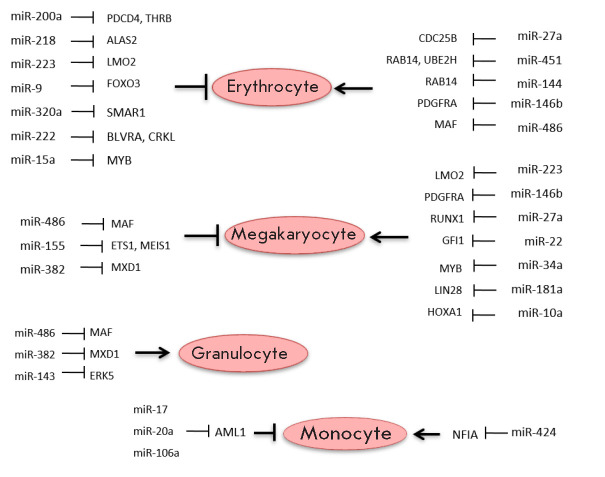
MiRNAs and their target genes involved in the regulation of hematopoiesis

Erythropoiesis is a process of CMP differentiation into a common megakaryocyte-erythrocyte progenitor, followed by the formation of erythrocytes. Erythrocyte dysplasia appears as a change in the shape of red cells; in particular, due to cytoskeletal abnormalities. A common concomitant pathology in MDS is anemia, associated either with a decrease in the number of erythrocytes or with a decrease in their hemoglobin levels. Analysis of literature data has shown that miRNAs control every step of hematopoiesis. Some miRNAs promote – while others block – the differentiation of precursors into mature blood cells (*Fig. 4*). In particular, increased expression levels of miRNA-200a, -218, -221, -222, -223, 9, -15a, and -320 block, while miRNA-27a, -451, -144, -486-3p, and -146b promote erythropoiesis [32-46]. In addition, decreased expression levels of miRNA-150 promote erythropoiesis [39]. Jin *et al. *note that the expression levels of miRNA-142-3p, miRNA- 142-5p, miRNA-146a, and miRNA-451 dynamically change during the differentiation of the erythroid lineage [40]. An interesting fact was noted by Sun *et al*.: a high-altitude hypoxic environment substantially increases the number of erythrocytes and influences the miRNA profiles of human erythrocytes. A substantial increase in expression levels was especially noted for miRNA-144-5p and miRNA-30b-5p [47].

Megakaryocytopoiesis occurs in bone marrow (BM) and is a multi-stage process whose final stage is platelet formation [48]. As was noted above, miRNA-451 promotes erythropoiesis; however, the expression level of this miRNA is decreased during the differentiation of megakaryocytes, indicating the decisive role of this miRNA at the stage of megakaryocyte-erythrocyte progenitor differentiation [49]. MicroRNA-150 acts similarly: its increased expression levels promote megakaryocytopoiesis; and the decreased levels – erythropoiesis [50]. Analysis of literature data has shown that increased expression levels of miRNA-223, -27a, -22, -146b, -34a, and -181a promote –and increased expression levels of miRNA-155, -486-3p, and 382-5p inhibit –the differentiation of megakaryocytes [35, 46, 51, 52, 53, 54, 55, 56]. In addition, the miRNA-10a expression is downregulated during megakaryocyte differentiation [57].

The formation of granulocytes and monocytes occurs because of the successive stages of differentiation, starting from CMPs. The morphological and functional abnormalities of granulocytes in part account for the bacterial infections in MDS. It is noted that miRNA- 486-3p promotes granulocyte differentiation and suppresses macrophage differentiation [45]. Increased expression levels of miRNA-223 promote granulopoiesis and block monocyte-macrophage differentiation [37]. In addition, increased expression levels of miRNA-143 and -382-5p are observed during granulocyte differentiation [56, 58]; increased miRNA-424 and decreased miRNA-17-5p, 20a, and 106a – during monocyte differentiation [59, 60, 61]. Rajasekhar *et al*. developed a miRNA profile of mature monocytes and granulocytes isolated from umbilical cord blood. These authors identified 46 miRNAs whose expression levels in both cell types were dissimilar to those of their CMPs. It is noteworthy that the miRNA-125b and miRNA-10a expression levels decreased 10- and 100-fold, respectively, in mature cells [62].

## MicroRNAs IN MDS

MicroRNAs are among the regulators of normal hematopoiesis, and it is not surprising that changes in their expression levels contribute to hematologic neoplasm development. Over the past ten years, several large-scale studies of MDS-specific miRNA expression profiles (*Table 1*) have been published [63-83]. However, only part of the results obtained aligned, because different authors worked with samples that were prepared differently and of different quality, while they also used different methods of analysis and statistical data processing.

**Table 1:** Differential miRNA expression in MDS

Material sampled	miRs with increased expression	miRs with decreased expression	Ref.
BM	miR-21, miR-720	miR-671-5p, miR-BART13	[63]
BM/MNC	miR-17-3p, miR-17-5p, miR-21, miR-155, miR-18a, miR-126, miR-181a, miR-10a, miR-10b, miR-15a, miR-16, miR-222		[64]
PB	miR-17-3p, miR-17-5p, miR-21, miR-18a, miR-15a, miR-142-3p		[64]
BM	miR-299-3p, miR-299-5p, miR-323-3p, miR-329, miR-665, miR-370, miR-409-3p, miR-431, miR-432, miR-494, miR-654-5p	miR-196a, miR-423-5p, miR-525-5p, miR-507, miR-583, miR-940, miR-1284, miR-1305	[65]
BM	miR-194-5p, miR-320a		[66]
BM		miR-378	[67]
BM		miR-93-5p	[68]
BM/MNC		miR-124a, miR-155, miR-182, miR-200c, miR-342-5p, let-7a	[69]
BM	miR-99a-5p		[70]
BM	miR-4462	miR-30d-5p, miR-222-3p, miR-30a-3p	[71]
BM	miR-661		[72]
BM/MNC		miR-124	[73]
BM/MNC	miR-636	miR-103, miR-140, miR-150, miR-342, miR-378, miR-483, miR-632	[74]
BM	miR-21		[75]
BM/MNC	miR-222, miR-10a, miR-196a, miR-320, miR-100	miR-124, miR-206, miR-326, miR-197, miR-875-5p, miR-146a, miR-150, let-7e	[76]
BM	miRNA-550a-5p		[77]
BM	miRNA-210 and miRNA-155		[78]
BM	miRNA-10a and miRNA-10b		[79]
Plasma		miRNA-16 and let-7a	[80]
Plasma	miRNA-150-5p	miRNA-16-5p, miRNA-27a3p, miRNA-199a-5p, miRNA-451a	[81]
BM	miRNA-205-5p		[82]
Plasma/vesicles	miRNA-10a-5p, miRNA-29a-3p, miRNA-34a-5p, miRNA-99b-5p, miRNA-125a-5p, miRNA-146b-5p and miRNA-150-3p/5p		[83]
Plasma	let-7a-3p, miRNA-21-3p, miRNA-221-3p, miRNA-221-3p/5p and miRNA-223-3p		[84]

BM – bone marrow,

PB – peripheral blood,

MNC – mononuclear cells.

Ozdogan *et al*. relate an interesting fact: in MDS, not only miRNA expression levels are changed, but also the expression of the *DICER1 *gene, a key participant in the canonical miRNA processing pathway, is decreased [71]. In particular, Jang *et al*. conclude that increased expression levels of miRNA-205-5p promote MDS by suppressing *PTEN *and, thus, acting as an oncogene in hematopoietic cells. In addition, increased expression levels of miRNA-205-5p are not associated with a decrease in the overall survival rate or with a certain prognostic group of MDS patients. This indicates that miRNA-205-5p is involved in the initiation, but not in the progression, of MDS [82]. Li *et al*. suggest that increased expression levels of miRNA10a/b are associated with myeloblast population growth [79].

MicroRNAs detected in the blood are referred to as “circulating miRNAs.” MicroRNAs are analyzed not only in the blood, but also in special structural elements named ‘exosomes’ that are nano-sized membrane vesicles that play an important role in the tumor microenvironment. It is noted that tumor cells release many more exosomes into the tumor microenvironment than normal cells do, leading to an increased level of exosomes in the circulatory system. The gene that promotes tumor growth can be transported by exosomes and promote metastasis. In particular, the miRNAs located in exosomes can contribute to oncogenesis [84]. Hrustincova *et al*. performed a unique comparative analysis of the expression levels of the miRNAs in total blood plasma and those of the miRNAs encapsulated in vesicles. They found that the populations of many hematopoiesis-associated miRNAs were substantially increased in MDS patients, mostly in both plasma and vesicles, although some miRNAs were unique to either plasma or vesicles. In addition, the expression levels of miRNA-103a-3p, -103b, -107, -221-3p, -221-5p, and miRNA-130b-5p were substantially decreased in the plasma of patients in a later stage of MDS compared to early-stage MDS patients. By contrast, the expression levels of miRNA-127-3p, -154-5p, -323b-3p, -382-3p, -409-5p, and miRNA-485-3p clustered in the chromosomal region 14q32 were increased at the early stage of MDS. The authors pointed out that certain profiles of miRNAs in plasma and vesicles appeared to represent two distinct biomarkers [83].

## MicroRNAs AND GENETIC CHANGES IN MDS

It has been shown repeatedly that a karyotype change correlates with a unique profile of miRNA expression (*Table 2*).

**Table 2:** Differential miRNA expression in karyotype-dependent MDS

Chromosomal aberration	miRs with increased expression	miRs with decreased expression	Ref.
del(5q)	miR-34a, miR-148a, miR-451, miR-486, miR-125a/b, miR-151, miR-199a, miR-10a/b, miR-29c, miR-130a, miR-24, miR-126, miR-335, miR-99b, miR-21, miR-17, miR-18a, miR-155	miR-128b, miR-95, miR-213, miR-520c, miR-146a, miR-449a, miR-300, miR-210, miR-193a-3p, miR-874, miR-589, miR-150, miRNA-143, miRNA-378, miR-145	[85]
monosomy 7/ del(7q)	miR-144, miR-451, miR-92a, miR-96, miR-340, miR-433, miR-105	miR-140-5p, miR-196b, miR-25, miR-590-3p, miR-511, miR-134	[85]
trisomy 8	miR-511, miR-146b, miR-134, miR-410, miR-153, miR-433, miR-105, miRNA-383	miR-10b, miR-452, miR-152, miR-181b, miR-28, miR-92, miR-10a, miR-324-3p, let-7a, miR-497, miR-24, miR-196b, miR-19a, miR-181c, miR-20a, miR-130b, miR-99a, miR-100, miR-515-3p, miR-199a	[85]
del(20q)	miR-206, miR-296-5p, miR-34b, miR-323-5p, miR-499-5p, miR-493, miR-503, miR-632, miR-98, miR-769-5p	miR-144, miR-451, miR-92a	[85]
monosomy 7/ del(7q)		miR-595	[86]
t(2;11)(p21;q23)	miRNA-125b-1		[87]
trisomy 1		miRNA-194-5p	[66]

Kuang *et al*. did their best to present as fully as possible the data obtained from the studies of correlations between miRNA expression levels and MDS-specific karyotypes [85]. Unbalanced chromosomal abnormalities are characteristic of MDS, and the most common are del(5q), monosomy 7 or del(7q), trisomy 8 and del(20q) [88]. Alkhatabi *et al*. showed that miRNA-595 expression levels are substantially decreased in MDS with -7/7q, as well as when a patient has a complex karyotype including chromosome 7 abnormalities [86]. Comparative analysis of miRNA expression levels in the presence of trisomy 1 demonstrated a decrease in the relative expression level of miRNA-194-5p in MDS patients with trisomy 1 compared to patients with the normal karyotype [66]. Another work provides data on the role of miRNA-150 in the MDS developing in del(5q) individuals. It was pointed out that this miRNA targets a MYB transcription factor for suppression and that its suppression promotes proliferation inhibition [89]. Fang *et al*., too, focus on the role of miRNAs in MDS developing in del(5q) individuals. It was demonstrated that MDS with this karyotype was characterized by an aberrant expression of more than 20 miRNAs, and most of them were located outside the deleted region 5q32 [90]. Analysis of the expression profile of 13 miRNAs located on 5q showed that the expression levels of miRNA-145 and miRNA-146a were substantially decreased in the BM cells of MDS patients with del(5q), as compared with the control group and patients with diploid karyotype [91]. However, Votavova* et al*. found that the expression levels of miRNA-378 and miRNA-146a were substantially decreased, and those of miRNA-34a were increased in del(5q) patients’ BM cells, while the expression levels of miRNA-143 and miRNA-145 were somewhat increased [92].

Balanced chromosomal rearrangements in MDS patients are rare. One of the chromosomal translocations in MDS is t(2;11)(p21;q23). Increased expression levels of miRNA-125b-1 that is located close to the chromosome 11 breakpoint provide additional support to the idea that changes in miRNA expression profiles are associated with fragile sites [87].

Analysis of the expression levels of the miRNAs located on chromosome 8 showed that trisomy 8 results in an increase of miRNA-383 expression only. This result indicates that no increase in ploidy entails an increase in most of the miRNAs on this chromosome, confirming the complexity of the miRNAs-mediated regulatory mechanisms of MDS initiation [90].

Mutations are an integral part of the genetic changes leading to MDS; in particular, mutations to the *SF3B1*,* SRSF2, *and *U2AF1 *genes involved in splicing are frequent in this disease [88]. It has been shown that the expression levels of let-7, miRNA-423, and miRNA-103a are decreased in MDS samples with mutations to these genes when compared with wild-type samples, suggesting the presence of complex molecular genetic cascades in MDS [93]. Analysis of the relationship between the presence of somatic mutations and the levels of circulating miRNAs in MDS demonstrated that the mutation to *Dnmt3a *was associated with changes in the expression levels of about 30 miRNAs in plasma and about 20 miRNAs in vesicles, and the presence of a mutation to *SF3B1*, with about 20 miRNAs expressing differentially in plasma and about 10 in vesicles, while only miRNA-100-5p and miRNA-450b-5 displayed unidirectional changes in expression levels, both in plasma and in vesicles [83].

## MicroRNAs AND MDS THERAPY

last decade has witnessed a breakthrough in MDS treatment. Three hypomethylating drugs have been approved therapeutically: azacitidine, decitabine, and lenalidomide. Nevertheless, it is still not always possible to achieve a proper response to the therapy [94]. A large number of works have been published seeking to analyze the correlations between miRNA expression levels and the response to the therapy offered in MDS. For example, analysis of miRNA-21 expression levels helps predict the response to hypomethylating agents and patients with low miRNA-21 expression levels in the serum had higher response rates [95].

Meng *et al*. noted that miRNA-124 expression levels are lower in MDS patients than in healthy donors, but that treatment with low doses of decitabine led to an increase in the expression in 7 out of 18 patients [73].

Analysis of miRNA expression levels in bone marrow before and during treatment with azacitidine showed that the response to the therapy was much better in patients with increased miRNA-17-3p and decreased miRNA-100-5p and miRNA-133b. Importantly, high expression levels of miRNA-100-5p at the beginning of the study were associated with a shorter overall survival rate. In addition, there was a decrease in the expression levels of miRNA-10b-5p, miRNA- 15a-5p/b-5p, miRNA-24-3p, and miRNA-148b-3p in responders [100]. Another study noted that analysis of the expression profiles of miRNA-423-5p, -126-3p, -151a-3p, -125a-5p, and miRNA-199a-3p in MDS patients’ plasma allowed one to predict their response to treatment with azacitidine [83].

Lenalidomide is an immunomodulatory and antiangiogenic drug used for treating del(5q) MDS. Interestingly, analysis of miRNA expression levels in bone marrow cells obtained from such patients showed that the miRNAs mapped to 14q32 were differentially expressing during treatment with lenalidomide [97]. It remains unknown whether the change in miRNA expression profiles is due to one of the actions of lenalidomide or simply a result of the abnormal clones’ population decline. In another study, analysis of miRNA expression levels in peripheral blood monocytes demonstrated a decrease in miRNA-34a-3p and miRNA-34a-5p expression levels, and an increase in miRNA-378-3p and miRNA-378-5p following exposure to lenalidomide compared to the expression levels before therapy [98]. Venner *et al*. pointed out that there was an increase in miRNA-143 and miRNA-145 expression levels following exposure to lenalidomide, noting their role in the response to the therapy offered [99]. Naming lenalidomide’s exact mechanism of action in MDS is important for oncohematology. However, there is not yet a diagnostic test that can predict the response to this drug. Thus, miRNAs are promising candidates to predict lenalidomide response.

## MicroRNAs AND MDS PROGNOSIS

The prognosis of the disease is assessed using the Revised International Prognostic Scoring System (IPSS-R), which divides patients into five risk subgroups, taking into account the karyotype, the number of blast cells, and the degrees of deficiency of various blood cell types. In particular, this prognostic system allows one to assess the overall survival rate and the risk of transformation to AML in MDS patients [100]. However, this system does not fully reflect the genetic complexity of this disease. In addition to the recognized predictors, miRNAs are some of the promising markers for making predictions of the outcome in MDS. MDS is regarded as a disease preceding leukemia, and about 30% of MDS patients eventually develop AML [11]. Analysis of literature data has shown that miRNA expression profiles differ between early and advanced stages of MDS, confirming the involvement of miRNAs in the pathogenesis of MDS and, consequently, in MDS-to-AML transformation (*Table 3*).

**Table 3:** MicroRNAs associated with disease progression

miRNAs	Material sampled	Expression levels in MDS	Implications	Ref.
miRNA-422a, -617, -181a, -222, and miRNA-210	BM	increased	disease progression	[85]
miRNA-196b-5p	BM	increased	risk of transformation to AML	[101]
miRNA-29b	BM	decreased	risk of transformation to AML	[102]
miRNA-125a	BM	increased	overall survival decreased	[103]
let-7a	BM	increased	overall survival decreased	[85]
miRNA-194-5p	BM	decreased	overall survival decreased	[66]
miRNA-22	BM	increased	overall survival decreased	[28, 104]
miRNA-661	BM	increased	overall survival decreased	[72]
miRNA-126 and miRNA-155	BM	increased	overall survival decreased without disease progression	[69]
miRNA-124а	BM	increased	overall survival decreased	[69]
miRNA-223-3p	plasma	decreased	overall survival decreased	[81]
miRNA-451	plasma	decreased	overall survival decreased without disease progression	[81]

BM – bone marrow.

Kuang *et al*. conducted an overview of data on the correlation of miRNA expression levels depending on the degree of disease progression. In particular, increased expression levels of miRNA-422a, -617, -181a, and miRNA-210 were associated with disease progression; increased expression levels of miRNA-17-5p, mRNA-20a, and miRNA-34a – with low-risk MDS [85]. In later studies, the understanding of the contribution of miRNAs to MDS was expanded. Wen *et al*. demonstrated that miRNA-196b-5p expression levels were increased in higher-risk MDS patients and in their peers who developed AML and proposed this miRNA as a biomarker associated with the risk of MDS transforming into leukemia [101]. In addition, Kirimura *et al*. emphasized that a decrease in miRNA-29b expression levels promoted the transformation of MDS into AML as well [102]. Choi *et al*. found that, according to IPSS, the expression levels of miRNA-21, miRNA-146b-5p, miRNA-126, and miRNA-155 were substantially higher in high-risk than in low-risk patients. Moreover, high expression levels of miRNA-126 and miRNA-155 correlated with a substantially lower overall survival rate and survival without transformation into leukemia, suggesting that these miRNAs could be associated with MDS progression and transformation to AML [69]. Alkhatab *et al*. determined that the expression level of miRNA-595 was lower in high-risk MDS; however, they emphasized that the data obtained required further research to involve a larger cohort of patients. In addition, this miRNA directly targeted the *RPL27A* gene and its downregulation disrupted erythropoiesis [88]. As was noted, miRNA-125a also contributed to impaired erythropoiesis, its expression was increased in MDS and negatively correlated with the overall survival rate of patients [103].

Some studies note correlations between miRNA expression levels and the chance of survival for MDS patients. In particular, decreased expression levels of miRNA-181 and miRNA-21 correlate with longer overall survival and increased expression levels of let-7a correlate with shorter patient survival [85]. In addition, low expression levels of miRNA-194-5p correlate with a decrease in the overall conditions of MDS patients [66].

Not only MDS patients’ BM cells, but also blood can be used as a source material, allowing one to identify miRNAs as readily available markers for the analysis of the development and prognosis of the disease. Analysis of circulating miRNAs in the plasma of MDS patients showed that the expression levels of miRNA-27a-3p, -150-5p, -199a-5p, -223-3p, and miRNA-451a were decreased in higher-risk MDS individuals. In addition, low expression levels of miRNA-451 were associated with decreased, progression-free survival rate; low expression levels of miRNA-223-3p, with a substantial decrease in the overall survival rate [81]. Zuo *et al*. presented a diagnostic panel including miRNA-144, -16, -25, -451, -651, -655, and let-7a, enabling one to select normal-karyotype patients based on the prognosis of survival [105].

Besides *de novo *MDS, cases of secondary MDS following treatment with cytostatic agents and characterized by a number of genetic changes identical to those in primary MDS are known in clinical practice. However, IPSS-R is focused only on primary MDSs. Secondary MDSs are heterogeneous as well and require the same careful classification into risk groups as primary MDSs; therefore, the search for prognostic markers in secondary MDSs, along with *de novo *MDSs, is an important line in hematology-oncology [88]. Very few analyses of miRNA expression levels in secondary MDS have been published. In particular, Le *et al*. showed that the expression levels of miRNA-99a-5p were higher in high-risk MDS patients and in patients with secondary MDS than in low-risk peers [70].

## MUTATIONS IN miRNA GENES AND MDS

Changes in miRNA expression levels and, as a consequence, the development of neoplasms are associated not only with mutations in protein-coding genes but also directly with mutations in miRNA genes. Thus, a study identified seven MDS patients with mutations in miRNA genes. All mutations were heterozygous, and most of them were located in the seed region of the gene encoding miRNA-142-3p. Mutations in the seed region lead to a gain of new target genes as well as to a loss of the target genes specific to this miRNA. Therefore, a conclusion can be made that the mutations affecting the seed region reduce the target specificity and provoke leucosis [106].

Similar to protein-coding genes, miRNA-encoding genes are regulated post-transcriptionally and this represents yet another mechanism for regulating miRNA expression levels, alongside mutations. In particular, the transcription factor TWIST-1 promotes an increase in miRNA-10a/b expression by binding directly to the promoters of the genes encoding these miRNAs and, thus, promoting the initiation of MDS [79]. Another example is miRNA-34a regulation by a transcription factor encoded by the tumor suppressor gene *p53*; in addition, it is noted that the expression levels of this miRNA are increased in patients with early-stage MDS [85].

Hypermethylation of miRNA promoters is another factor of MDS progression. In particular, the miRNA-34b gene promoter was found hypermethylated in MDS patients and this status had probably been acquired during progression to AML [85]. In addition, hypomethylation of the let-7a-3 and miRNA-124-3 gene promoters correlates with a poor survival chance of MDS patients and a poor prognosis of the disease [107, 108].

## CONCLUSION

Molecular genetic markers are gradually becoming more and more popular in describing MDS; in addition, they discriminate MDS from other BM conditions [109, 110, 111, 112, 113]. As described above, unique miRNA expression profiles are characteristic of different MDS subtypes. Nevertheless, further research is needed to understand the complex regulatory mechanisms operating between miRNAs and their target genes in MDS. Most of the works are based on the analysis of miRNA expression levels in BM or peripheral blood. Unlike mRNAs, miRNAs are highly stable, allowing the BM material embedded in paraffin or fixed on coverslips to be accessible to the analysis.

It should be admitted that a universal prognostic scoring system covering all important MDS parameters has yet to be developed. Therefore, an important task before clinical oncology is to search for additional molecular-genetic markers that can be integrated into the existing international prognostic systems, and some of the most promising candidates for that role are miRNAs. Current data on the roles of miRNAs in MDS suggest that these molecules have the potential to become tools for the diagnosis and prognosis of MDS and may be relevant to the response to treatment.

In addition to using miRNAs in diagnostic and prognostic tasks, one of the promising avenues in scientific research is trying them out as therapeutic targets. MicroRNA-mimics-34 encapsulated in lipid nanoparticles is the most studied potential therapeutic agent for the treatment of lung cancer. In addition, several preclinical studies have explored an antitumor strategy based on the suppression of oncomiRNAs with the use of antisense oligonucleotides (anti-miRNAs). In MDS, a chemically modified inhibitor of miRNA-21 promotes normal erythropoiesis and increases hematocrit [114].

In conclusion, cases of secondary MDS associated with morphological BM cell abnormalities and imbalance between different blood cell types, similar to those in primary MDS, are known in practice. It is possible that secondary MDS develops after cytostatic therapy or in patients with autoimmune diseases, solid tumor neoplasms, some infections, and other pathologies accompanied by secondary hematopoietic conditions. Of special interest are MDS in untreated patients with malignant lymphomas. An important discovery is the fact that NHML patients with signs of erythroid lineage dysplasia develop anemia about twice as often as their peers with morphologically normal BM cells, and collectively these facts are associated with rapid tumor progression, a low three-year survival rate, and resistance to the treatment offered [115].

Thus, the search for additional prognostic markers for the diagnosis of both *de novo *MDS and secondary lesions to BM will allow us to develop personalized treatment that is as precise as possible.
